# Commentary on ‘Management of anastomotic leaks after elective colorectal resections: The East of England experience. A retrospective cohort’ [Int J Surg 2021; 96:106167]

**DOI:** 10.1097/JS9.0000000000000616

**Published:** 2023-08-17

**Authors:** Zengwu Yao, Jinchen Hu, Lixin Jiang

**Affiliations:** a Yantai Yuhuangding Hospital, Shandong University; b Department of Gastrointestinal Surgery, Yantai Yuhuangding Hospital affiliated with Qingdao University; c General Surgery, Yantai Yeda Hospital, Shandong Province, People’s Republic of China

*Dear Editor*,

We were very interested in the article by Aker *et al*.^1^ titled, ‘Management of anastomotic leaks after elective colorectal resections: The East of England experience.’ In this retrospective cohort study, 201 (5.9%) of 3391 elective colorectal resections across seven hospitals with confirmed anastomotic leaks were included. This multicenter study aimed to investigate the different methods of managing anastomotic leaks and their effectiveness and outcomes. The results showed that despite initial conservative, antibiotic, and radiological interventions being successful in the majority of patients, two out of five patients still required laparotomy, and over a quarter of patients had an end stoma. We thank the authors for providing valuable data for this study; however, we would like to make several comments.

Despite the significant progress in surgical techniques, anastomotic leakage in colorectal surgery still plagues surgeons. Unsuccessful management can lead to severe abdominal infections, sepsis, and even death. Therefore, the management of anastomotic leakage remains a very important aspect of perioperative management. The Italian ColoRectal Anastomotic Leakage (iCral) Study Group reported similar results^2^. Among the treatment methods reported by the authors, the proportion of patients who received conservative management was lower (6.6% vs. 54.2%). This may be related to the definition of anastomotic leakage; it is possible that some anastomotic leaks classified as Grade A were not included in the analysis, leading to a significant reduction in the proportion of patients receiving conservative management. In addition, the high-risk factors related to mortality were age and anastomotic breakdown. This is concerning because the mortality rates reported in the two articles were similar (10.5% vs. 8.1%). However, the article by Aker *et al*. did not provide a detailed explanation of the initial treatment from which these deaths occurred or the specific cause of death. In order to identify factors possibly related to the choice of anastomotic leakage management, sex, age, nutritional status, American Society of Anesthesiologists (ASA) class, and perioperative blood transfusion were included in the analysis of the iCral Study Group. However, although this was a retrospective study, Aker *et al*. did not include a sufficient number of factors. This may exclude many key factors, such as the patient’s nutritional status, preoperative neoadjuvant radiotherapy, chemotherapy, etc.

Second, the intraoperative operation affects the severity and treatment of postoperative anastomotic leakage. For example, measures such as preserving the left colonic blood vessels, suturing and reinforcing the anastomotic stoma, closing the dissected peritoneum by suturing the colon and mesentery, and placing a transanal drainage to reduce the probability of a second laparotomy in low anterior resection for rectal cancer^3^. Although such data are difficult to obtain, they influence the approach to the management of anastomotic leakage.

Third, transpelvic, transanal, and radiological procedure drainage methods are commonly used. However, active drainage was more effective than passive drainage^4^. Therefore, at our center, we routinely perform continuously irrigated triple-lumen drainage in the pelvis for immediate, continuous irrigation in the event of an anastomotic leak (Grade B)^5^. Unpublished data from our center show that approximately two-thirds of Grade B leaks avoid secondary surgery by using this method.

Despite the above concerns, this is still a valuable study that retrospectively analyzes the effectiveness of different methods for managing anastomotic leakage in large cases from multiple centers.

## Ethical approval

Not applicable.

## Consent

Not applicable.

## Sources of funding

Shandong University Cooperation Project (3460019005).

## Author contribution

Z.Y.: writing the paper; Z.Y., J.H., and L.J. study concept or design; Z.Y. and J.H.: data collection and data analysis.

## Conflicts of interest disclosure

The authors declare that they have no conflicts of interest.

## Research registration unique identifying number (UIN)

Not applicable.

## Guarantor

Lixin Jiang.

## Provenance and peer review

Commentary, internally reviewed.

## Data availability statement

Not applicable.
